# Intra-Abdominal Gossypiboma: A Rare Cause of Palpable Abdominal Mass With a Review of Literature

**DOI:** 10.7759/cureus.10930

**Published:** 2020-10-13

**Authors:** Hazem El Zemity, Naif Hakami, Mohammed Abdelnour A Alfaki, Mohammed Khurizi, Ahmad Al-Zahrani

**Affiliations:** 1 Surgery, King Fahad Hospital, Albaha, SAU

**Keywords:** gossypiboma, textiloma, retained foreign object (rfo)

## Abstract

Gossypiboma is a term used to describe a pseudotumor caused by accidental retention of surgical swab or sponge in the body after surgery. The abdominal cavity is the most common site of retained surgical sponge. It is quite an infrequent surgical complication which is usually rarely reported because of the fear of medico-legal consequences. Here, we are reporting a case of a 26-years-old woman referred to our outpatient surgery clinic (OPD) from another hospital with complaint of intermittent abdominal pain, fever, and abdominal lump for 4 months following removal of IUD, which was attempted laparoscopically, and later converted to open laparotomy. She also had a history of cesarean section done one and half year ago in the same hospital. Clinical examination revealed a palpable abdominal mass in the para-umblical region. However, a computed tomography (CT) revealed a huge intra-abdominal mass. A diagnosis of intra-abdominal gossypiboma was suggested and the patient underwent exploratory laparotomy where the diagnosis was confirmed and the mass was excised.

## Introduction

Gossypiboma, textiloma or more broadly retained foreign object (RFO) is the technical term for surgical complications resulting from foreign materials, such as a surgical sponge, accidentally left inside a patient's body. Gossypiboma is a mass formed around involuntarily retained surgical sponge or swap after surgery due to foreign body reaction. The term is derived from a Latin word “Gossypium” that is cotton wool and the suffix word - oma” that means a tumor or growth, and describes a mass within a patient's body comprising a cotton matrix surrounded by a foreign body granuloma [1]. It’s quite an infrequent surgical complication which is usually rarely reported because of the fear of medico-legal consequences. The incidence of retained foreign bodies following surgery has a reported rate of 0.01% to 0.001%, of which Gossypiboma make up 80% of cases [2]. Because the clinical presentation of Gossypiboma is usually non-specific and it may be silent for months or even many years, the diagnosis usually needs a high index of suspicion. Various imaging modalities are present such as plain radiography, ultrasonography (USG), computed tomography (CT), and magnetic resonance imaging (MRI) that can help in making the diagnosis [3]. However, the CT findings of Gossypiboma, particularly in long-standing cases, may be indistinguishable from an intra-abdominal abscess, since air bubbles and calcification of the cavity wall as well as contrast enhancement of the rim may be seen in both the conditions [4]. Gossypiboma can be hard to diagnose specially in a later presentation. However, in patients with abdominal mass and history of abdominal surgical operation, the diagnosis of intra-abdominal Gossypiboma should keep in mind.

## Case presentation

**Figure 1:**
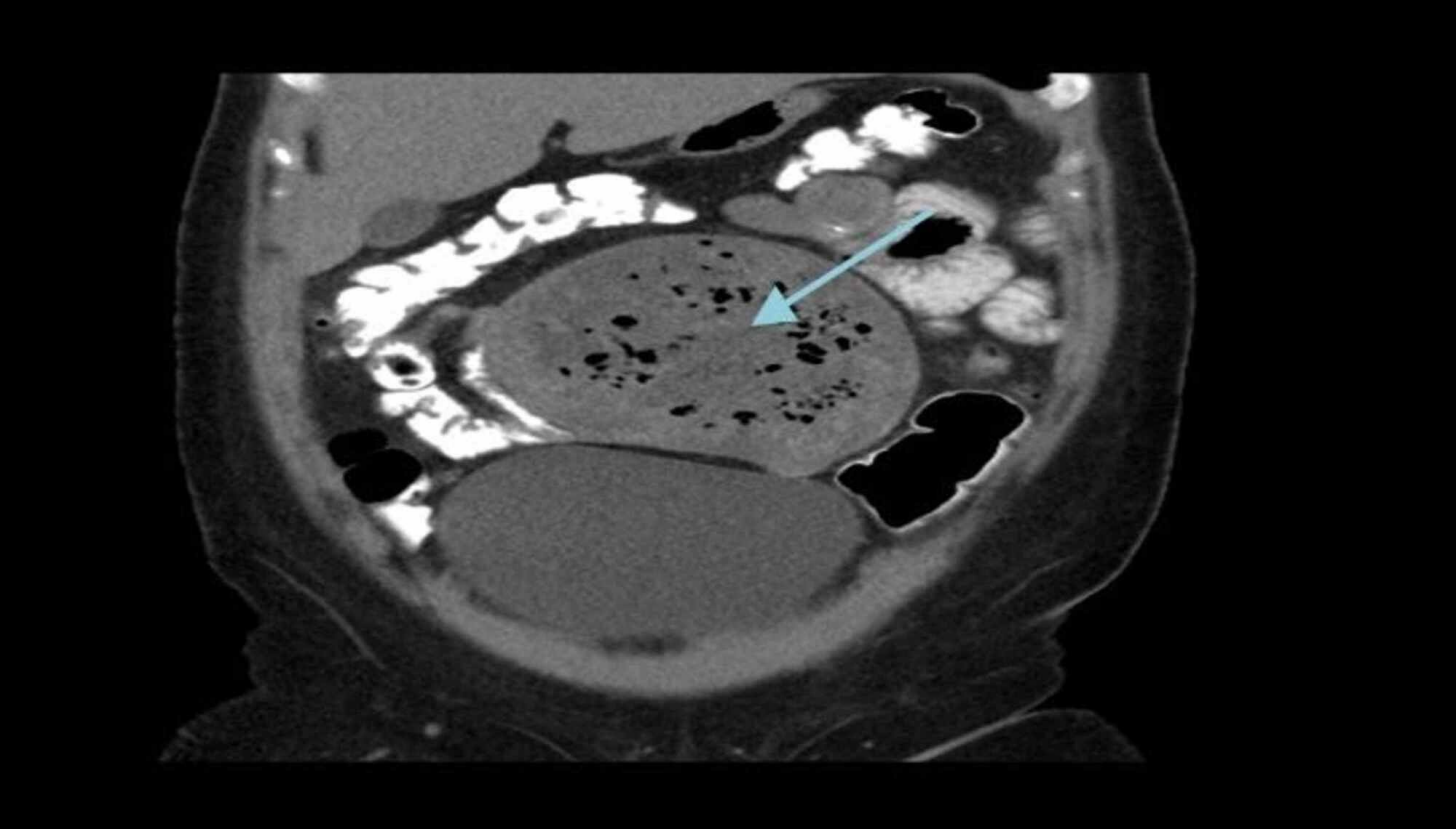
CT Abdomen showing huge intra-abdominal mass.

**Figure 2:**
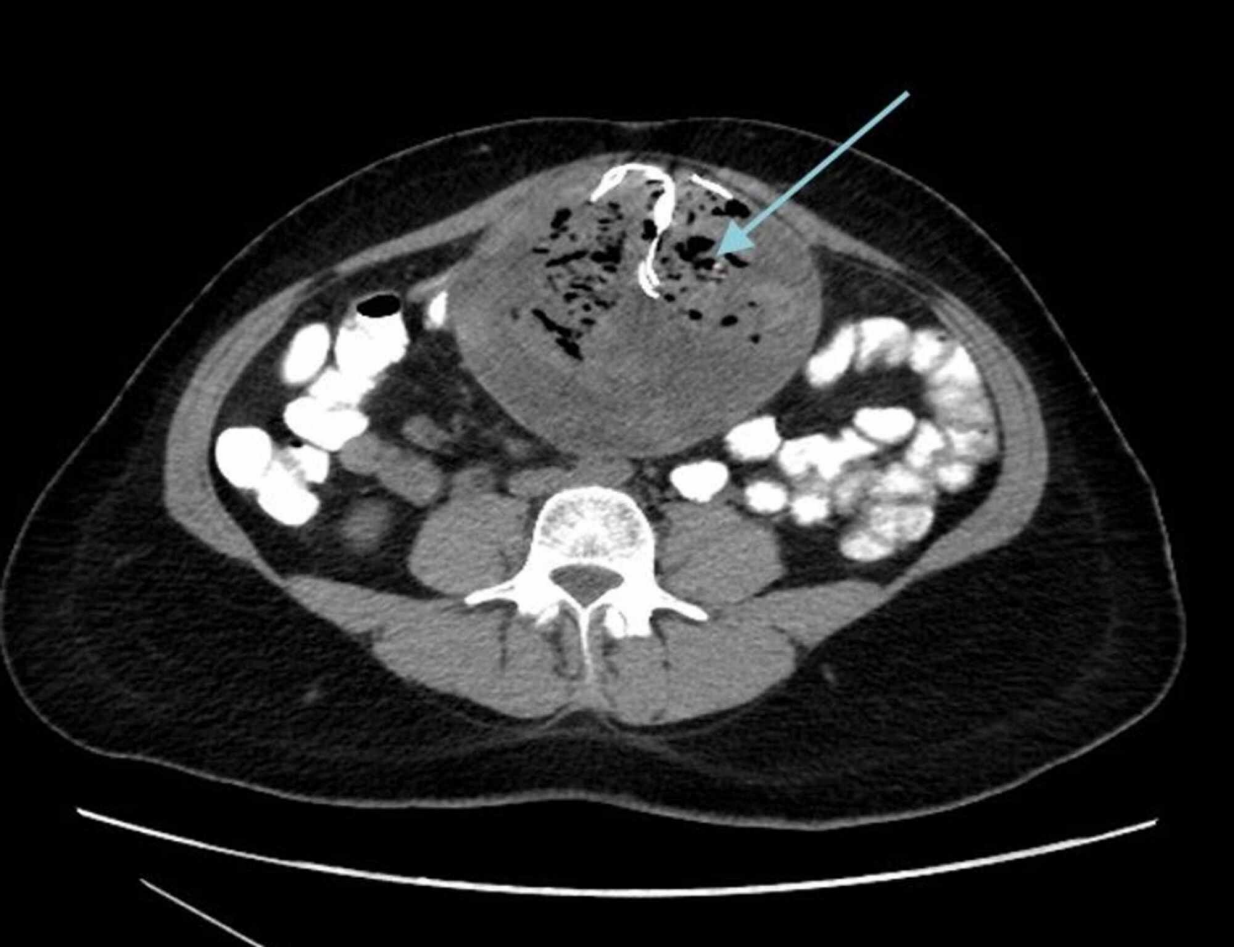
CT Abdomen showing intra-abdominal mass congaing air bubbles and hyperdense linear structure.

**Figure 3:**
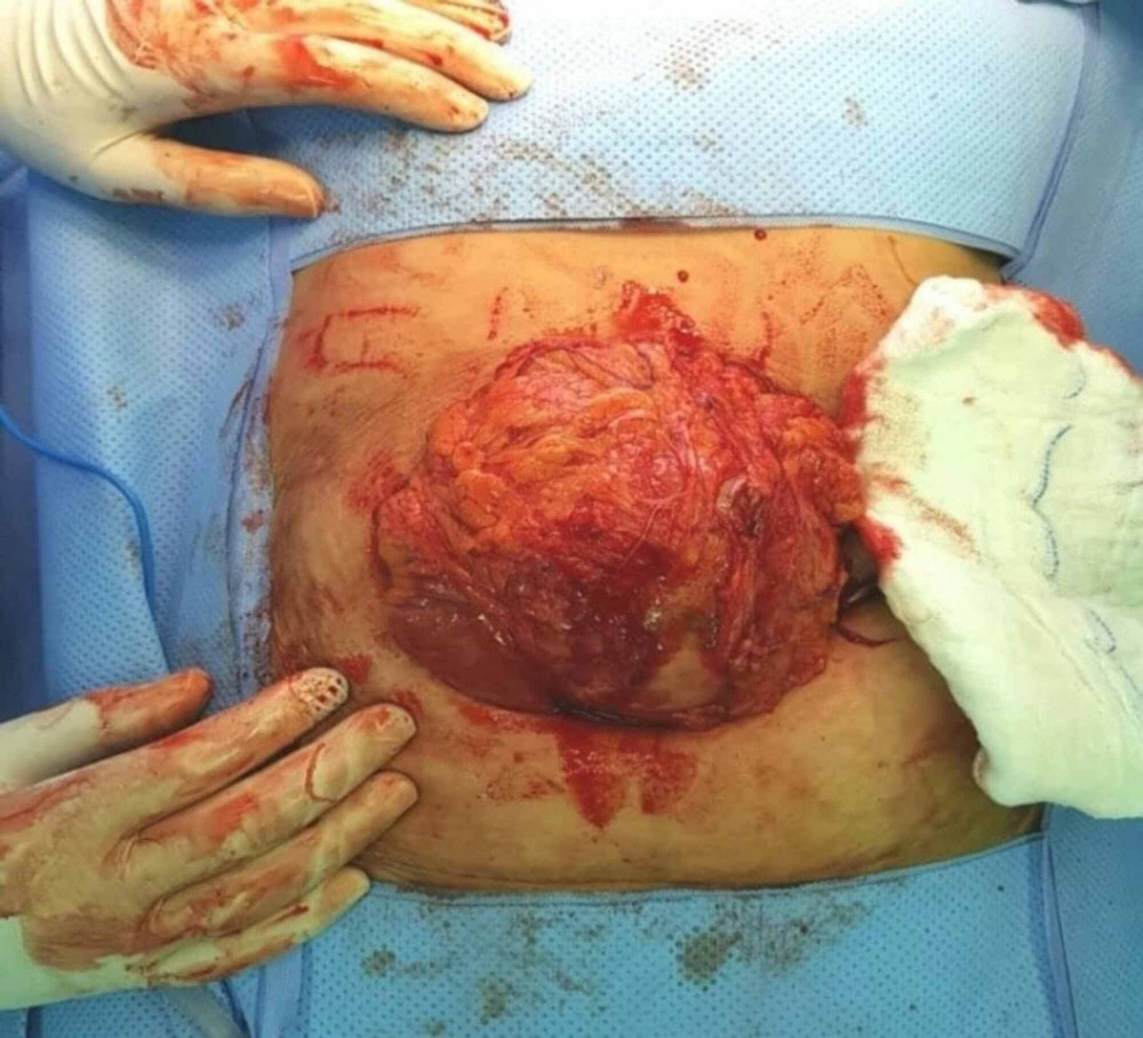
Intra-operative picture showing the lump densely adherent to the omentum.

**Figure 4:**
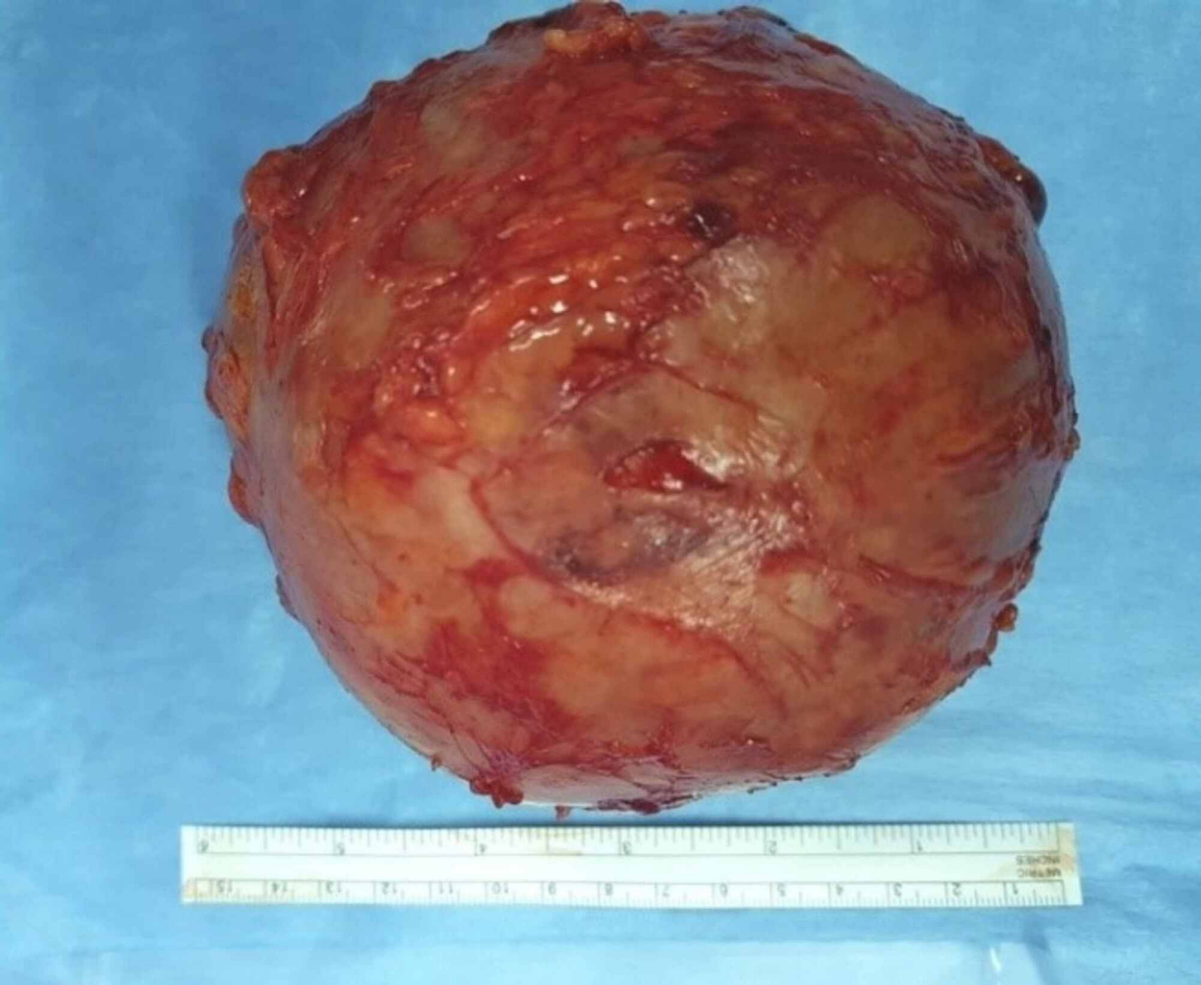
Picture showing the resected surgical specimen (Gossypiboma).

**Figure 5:**
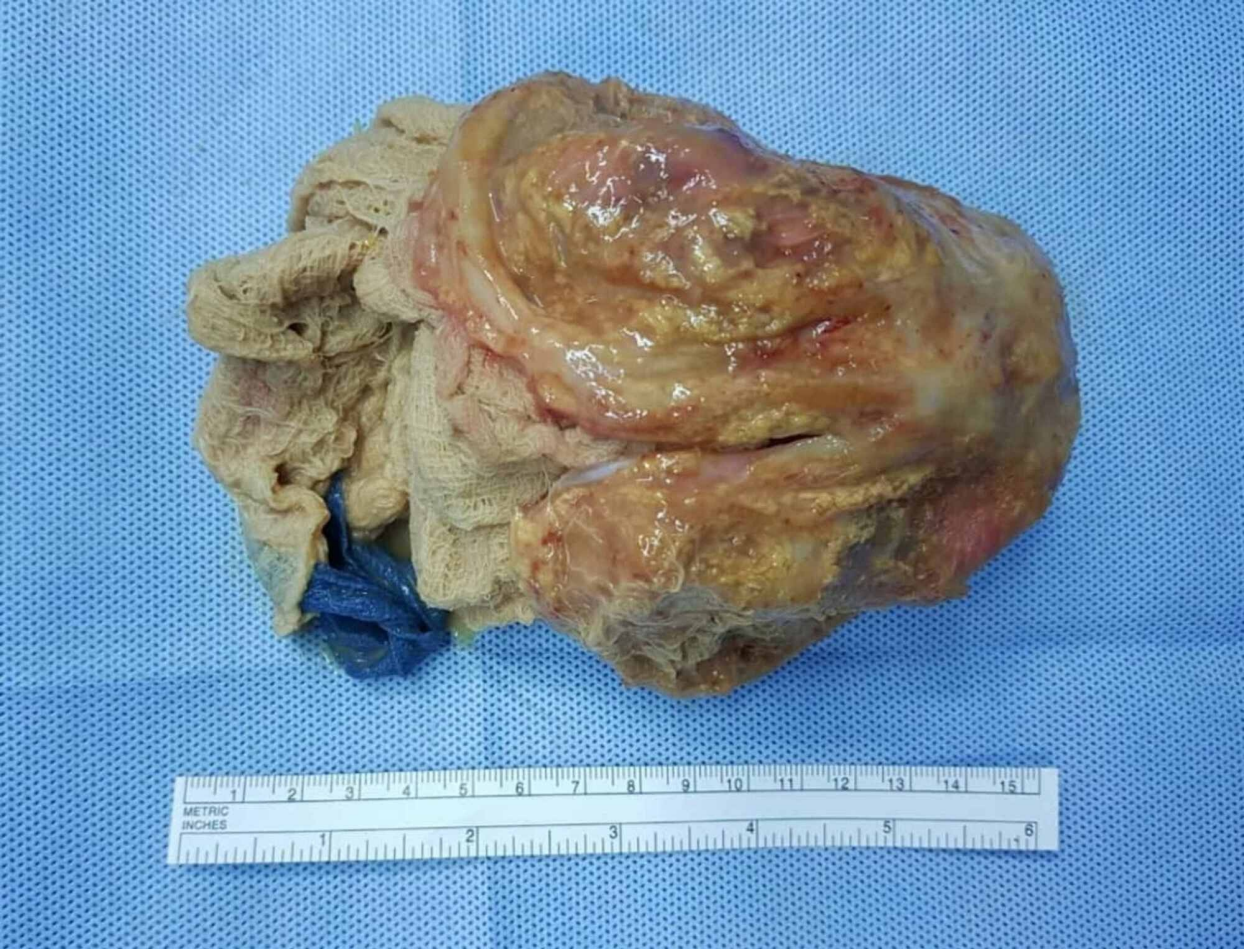
Picture of an opened resected surgical specimen containing laparotomy pad filled with pus.

A 26 year old woman presented to our hospital with complaints of intermittent abdominal pain, fever an abdominal lump for the past two months. She had history of elective caesarean section done one and half year ago in the referring hospital. Six months later, she underwent surgical removal of IUD in same hospital due to IUD migration; the operation was started laparoscopically and later converted to open due to technical difficulties and adhesions. Three weeks after the operation, the patient developed abdominal pain, high grade fever reaching up to 38^o^C, and purulent fluid discharge from the site of surgery. The patient was reassured and discharged on oral antibiotic. During the following months her condition did not improve and she had recurrent episodes of vague abdominal pain. However, within the past two months, the pain increased in intensity and the patient started to notice an abdominal lump which was progressively increasing in size. The patient was then referred to our hospital. Physical examination revealed palpable abdominal mass in the para-umblical region, measuring 16cm x 14cm in size, rounded, firm in consistency, and mildly tender. All routine investigations were within normal limits. Contrast enhanced computed tomography (CT) revealed well defined heterogeneous intra-abdominal mass measuring 15 cm x 14 cm x 12 cm, located in the umbilical region, showing enhanced wall (Figure 1). The mass contained multiple air bubbles, and had a hyperdense linear structure (Figure 2). A suggested diagnosis of intra-abdominal Gossypiboma was made and the patient was taken up for elective exploratory laparotomy. Intra-operatively, the mass was found to be densely adherent to the small bowel and omentum (Figure 3). Meticulous adhesiolysis was done and the mass safely resected (Figure 4). Upon opening, the mass showed retained lap pad with thick pus (Figure 5). No bowel resection was required. The postoperative period went uneventfully.

## Discussion

Although Gossypiboma or retained foreign body (RFB) is a quite rare surgical complication, it can lead to serious complication and increase in the mortality and morbidity rate. The inflammatory reaction to the non-absorbable cotton sponge leads to a variety of costs to society, including pain and emotional distress experienced by the patient, imaging studies to diagnose the problem, a subsequent hospitalization for reoperation, litigation expenses and patient compensation for perceived negligence of the facility and staff [5]. The true incidence of intra-abdominal Gossypiboma is not precisely known because it's seldom reported due to the medico-legal repercussions. However, it is estimated that Gossypiboma may occur in 1 out of 300-1000 of all surgical interventions and 1 out of 1000-1500 of intra-abdominal operations [2]. Numerous reports about Gossypiboma have been published up to date in the literature since it was described by Wilson in 1884. However, the increasing number of recent reports in the literature implies that this issue still remains as an important problem to be solved after intra-abdominal surgery [6].

The possible causes of sponge retention are emergency surgery, unexpected change in the surgical procedure, disorganization (e.g. poor communication), hurried sponge counts, long operations, unstable patient condition, inexperienced staff, inadequate staff numbers, and obesity [7]. The three significant risk factors were emergency surgery, unplanned change in the operation, and obesity. In addition, in 88% of the cases where there was a RFB and counts were performed, the counts were falsely called correct [8]. Intra-abdominal Gossypiboma can have various clinical presentations.

The reported cases in literature have shown that some cases may remain silent and not detected for many years after surgery. However, the main signs and symptoms are abdominal pain (42%), palpable abdominal mass (27%), and fever (12%) [9]. Less commonly, patients may present with complications including fistula formation, intestinal obstruction or bowel perforation. Gossypiboma can be difficult to diagnose in radiography and often confused with abscess, tumor, or a hydrated or mesenteric cyst. Imaging modalities including ultrasonography (USG) and Computed Tomography (CT) may help in the diagnosis. Characteristic finding on CT includes heterogeneous mass, enhanced wall, multiple air foci and hyperdense linear structure (radiopaque strip marker). In our case, all these were clearly shown on CT. Once a diagnosis of Gossypiboma is suspected, surgical removal is the recommended treatment. However, it is an avoidable surgical complication, and therefore strict measure should be applied to prevent it. The currently recommended operating room nursing procedure requires three separate counts of potential foreign bodies: once before the surgery, then again during the surgery, and finally once the incision is closed [7].

## Conclusions

Gossypiboma or retained surgical swap can be difficult to diagnose due its various and vague clinical presentations. However, in patients who present with palpable abdominal mass and a history of previous abdominal surgical operations, the diagnosis of Gossypiboma should be included in the differential diagnosis. Even though it’s a rare condition, it can cause serious complications and has medico-legal implications. Therefore, strict measures should be implemented to avoid it.
